# Antifeedant, antifungal and nematicidal compounds from the endophyte *Stemphylium solani* isolated from wormwood

**DOI:** 10.1038/s41598-024-64467-w

**Published:** 2024-06-12

**Authors:** Carmen E. Diaz, Maria Fe Andres, Rodney Lacret, Raimundo Cabrera, Cristina Gimenez, Nutan Kaushik, Azucena Gonzalez-Coloma

**Affiliations:** 1 Instituto de Productos Naturales, y Agrobiologia, Consejo Superior de Investigaciones Cientificas, Avda. Astrofisico F. Sanchez 3, 38206 La Laguna, Tenerife Spain; 2 Instituto de Ciencias Agrarias, Consejo Superrior de Investigaciones Cientificas, Serrano 115, 28006 Madrid, Spain; 3 University of La Laguna, 38206 La Laguna, Tenerife Spain; 4 The Amity Food and Agriculture Foundation, Amity University, Noida, Uttar Pradesh 201313 India

**Keywords:** Biotechnology, Chemical biology

## Abstract

The continuous search for natural product-based biopesticides from fungi isolated from untapped sources is an effective tool. In this study, we studied a pre-selected fungal endophyte, isolate Aa22, from the medicinal plant *Artemisia absinthium*, along with the antifungal, insect antifeedant and nematicidal compounds present in the extract. The endophyte Aa22 was identified as *Stemphylium solani* by molecular analysis. The antifungal activity was tested by broth microdilution against *Fusarium solani, F. oxysporum, F. moniliforme* and *Botrytis cinerea*, the insect antifeedant by choice bioassays against *Spodoptera littoralis, Myzus persicae* and *Rhopalosiphum padi* and the in vitro mortality against the root-knot nematode *Meloiydogyne javanica*. The structures of bioactive compounds were determined on the basis of 1D and 2D NMR spectroscopy and mass spectrometry*.* The ethyl acetate extract obtained from the solid rice fermentation showed mycelial growth inhibition of fungal pathogens (EC_50_ 0.08–0.31 mg/mL), was antifeedant to *M. persicae* (99%) and nematicidal (68% mortality). A bioguided fractionation led to the isolation of the new compound stempholone A (**1**), and the known stempholone B (**2**) and stemphol (**3**). These compounds exhibited antifeedant (EC_50_ 0.50 mg/mL), antifungal (EC_50_ 0.02–0.43 mg/L) and nematicidal (MLD 0.5 mg/mL) activities. The extract activities can be explained by **3** (antifungal), **1**–**3** (antifeedant) and **1** (nematicidal). Phytotoxicity tests on *Lolium perenne* and *Lactuca sativa* showed that the extract and **1** increased *L. sativa* root growth (121–130%) and **1** reduced *L. perenne* growth (48–49%). These results highlight the potential of the endophytic fungi Aa22 as biotechnological source of natural product-based biopesticides.

## Introduction

Endophytes are asymptomatic microorganisms that live in the host plant^1^. The host plant has increased resistance to herbivores, pathogens, and stress when colonized by endophytic microorganisms^2^. Endophytes can stimulate plant growth by producing phytohormones. Endophytes can inhibit plant pathogens by antagonistic action, by stimulating induced plant systemic resistance or by the action of secondary metabolites. This is why endophytes are a potential source of novel natural products of value in medicine, agriculture, and industry^1,3–6^.

Sometimes, these molecules can be found in the host plant and therefore the selection of host plants producing bioactive secondary metabolites has been suggested as criteria for the isolation of fungal endophytes with the ability to produce bioactive secondary metabolites^7,8^.

In recent years, our studies have been focused on the assessment and valorization of the endophytic fungal biodiversity from medicinal plants for the production of fungal biopesticides. One of such plants is wormwood (*Artemisia absinthium*), a medicinal plant known from ancient times that produces essential oil, bitter sesquiterpenoid lactones, flavonoids and lignans, among others and has antiprotozoal, antibacterial, antifungal, anti-ulcer, hepatoprotective, anti-inflammatory, immunomodulatory, cytotoxic, analgesic, neuroprotective, anti-depressant, neurotrophic, cell membrane stabilizing and antioxidant effects^9^. Furthermore, endophytic bacteria from *A. absinthium* plants are a remarkable source of antimicrobial and anticancer compounds such as 2-aminoacetophenone, 1,2-apyrazine-1,4-dione, phenazine and 2-phenyl-4-cyanopyridine^10^.

Our previous studies on a Spanish population of *A. absinthium* showed strong antifungal and anti-protozoan effects for the plant essential oil, with (-)-*cis*-chrysanthenol being identified as the chemical indicator of the antifungal effect. The antiprotozoal activity of the oil was related to the presence of *trans*-caryophyllene and dihydrochamazulene^11^. The study of the insect antifeedant effects showed that overall, the *A. absinthium* essential oil had moderate-low insect effects, while *Leptinortarsa decemlineata* was strongly affected by the ethanolic extract, characterized by the presence of the sequiterpene hydroxypelenolide, and flavones artemetin and casticin^9^. The sustainable production of this thujone-free plant population has been made possible through its domestication and variety registration (Candial^®^)^11,12^.

Therefore, in the present study, the plant *A. absinthium* (var. Candial) was chosen for the isolation of fungal endophytes to study their ability to produce additional biopesticidal metabolites (antifungal, nematicidal and insect antifeedant agents) to the ones found in the plant, as part of a larger project on the study of fungal endophyes under a collaborative Indo-Spanish framework (CDTI Spain and DST India).

**Figure 1:**
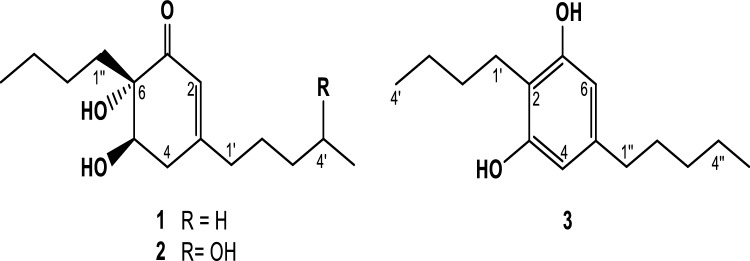
Compounds **1**–**3** isolated from *Stemphylium solani*.

The endophytic fungal strain Aa22 was isolated from leaves of *A. absinthium* and identified. Aa22 was fermented on rice and extracted with ethyl acetate (EtOAc) to give an extract with plant protection properties. The bioassay-guided fractionation of the extract gave three active compounds (**1**–**3**) that have been identified based on their spectroscopic data (Fig. 1). The extract and pure compounds were tested against fungal pathogens (*Fusarium solani, F oxysporum, F moniliforme* and *Botrytis cinerea*) insect pests (*Spodoptera littoralis, Myzus persicae* and *Rhopalosiphum padi*) and the plant parasitic nematode *Meloiydogyne javanica*. Detailed findings regarding the active metabolites and their plant protection potential are presented in this paper.

## Results

In this study the endophytic fungi Aa22, isolated from leaves of *Artemisia absinthium,* has been identified by molecular analysis as as *Stemphylum solani*. The EtOAc extract from the rice fermentation of this strain was tested against fungal pathogens (*Fusarium moniliforme, F. solani* and *Botrytis cinerea*), insect pests (*Spodoptera littoralis, Myzus persicae* and *Rhopalosiphum padi*), the root-knot nematode *Meloidogyne javanica* and seeds of *Lolium perenne* and *Lactuca sativa*. The extract was antifungal to *Fusarium* (*F. solani* > *F. moniliforme*) and *B. cinerea* with effective concentration (EC_50_) values of 0.2, 0.1 and 0.3 mg/mL, respectively. The extract was active on the aphid *M. persicae* (EC_50_ value of 6.0 gμ/cm^2^) and moderately affected the nematode *M. javanica* (68% J2 mortality). Additionally, the extract increased *L. sativa* root growth (121% increase).

This bioactive extract was submitted to a bio-guided chemical study (data in Tables S1 and S2 of Supplementary Materials) to afford stempholone A (**1**), stempholone B (**2**) and stemphol (**3**). (Fig. 1). Their structures have been elucidated on the basis of their spectroscopic data.

Compound **1** was isolated as an amorphous brown solid. The molecular formula was determined as C_15_H_26_O_3_ from the molecular ion peak at *m*/*z* 254.1888 (Calcd. 254.1882) in the HREIMS. The IR absorptions at 3443, 1673, and 1650 cm^–1^ indicated the presence of hydroxyl groups, carbonyl group and double bonds, respectively. The ^1^H NMR of compound **1** (Table 1) revealed signals characteristic of *n*-alkyl chains with two methyl groups at δ 0.85 (3H, *t,* J = 7 Hz, H-5´) and δ 0.88 (3H, *t,* J = 7 Hz, H-5´´) and an integral of 20 protons in total. Other signals that appeared in the proton resonance spectrum of **1** were assigned to one methylene at δ 2.47 (1H, *ddd*, *J* = 18.0, 10, 2.7 Hz, H-4ax) and δ 2.61 (1H, *dd*, *J* = 18.0, 6.0 Hz, H-4ec), one methine at δ 3.99 (1H, *dd*, *J* = 10.0, 6.0 Hz, H-5) bonded to oxygen and a signal of olefinic proton at δ 5.89 (1H, *bs*, H-2). Additionally, two triplet of doublets at δ 2.23 (2H, td, *J* = 7.0, 3.0 Hz, H-1´) and 1.95 (1H, td, *J* = 13.0, 4.0 Hz, H-1´´), along with a multiplet at δ 1.45 (1H, m, H-1´´) corresponding to the protons of two methylene groups, were observed.

**Table 1:** NMR spectroscopic data (500 MHz, CDCl3) for compounds **1** and **2**.

Position	1		2	
	δ_C_, type	δ_H_ (*J* in Hz)	δ_C_^a^, type	δ_H_ (*J* in Hz)
1	201.3, C		201.3, C	
2	122.5, CH	5.89, bs	122.6, CH	5.91, bs
3	164.2, C		163.6, CH	
4	36.1, CH_2_	2.47, ddd (18.0, 10.0, 2.7) 2.61, dd (18.0, 6.0)	36.0, CH_2_	2.47, ddt (18.0, 10.0, 2.7) 2.63, dd (18.0, 6.0)
5	73.9, CH	3.99, dd (10.0, 6.0)	73.8, CH	3.98, dd (10.0, 6.0)
6	79.6, C		79.6, CH	
1’	37.7, CH_2_	2.23, td (7.0, 3.0)	37.6, CH_2_	2.27, m
2’	26.7, CH_2_	1.52, m	23.2 (23.2), CH_2_	1.57, m 1.68, m
3’	31.3, CH_2_	1.28, m	38.6 (38.5), CH_2_	1.46, m
4’	22.4, CH_2_	1.32, m	67.7 (67.6), CH	3.82, q (6.0)
5’	13.9, CH_3_	0.88, t (7.0)	23.8 (23.7), CH_3_	1.20, d (6.0)
1’’	29.3, CH_2_	1.45, m 1.95, td (13.0, 4.0)	29.2, CH_2_	1.45, m 1.95, td (13.0, 4.0)
2’’	24.5, CH_2_	0.93, m 1.37, m	24.5, CH_2_	0.94, m 1.37, m
3’’	23.0, CH_2_	1.28, m	23.1, CH_2_	1.28, m
4’’	13.9, CH_3_	0.85, t (7.0)	13.9, CH_3_	0.85, t (7.0)

The ^13^C-NMR spectrum (Table 1) showed the presence signals of 15 carbons attributed by HSQC experiment to two methyls at δ 13.9 (C-4´´ and C-5´´), eight methylene carbons, two methine groups, one olefinic at δ 122.5 (C-2) and a carbon bonded to oxygen at δ 73.9 (C-5), together with those three quaternary carbons. One of them, bearing an oxygen function at δ 79.6 was assigned to C-6, whilst signals at δ 164.2 (C-3) and δ 201.3 (C-1) were consistent with the presence of carbon of trisubstituted double bond and a carbonyl group, respectively.

**Figure 2:**
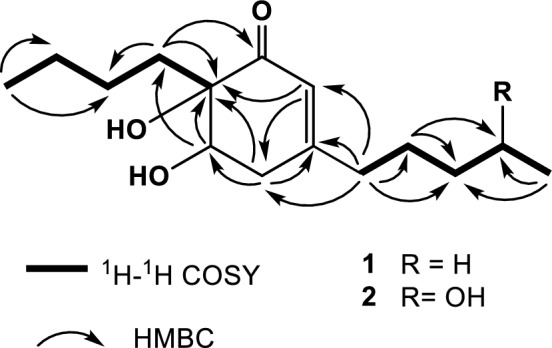
^1^H-^1^H COSY and HMBC correlations for compounds **1** and **2**.

From the ^1^H-^1^H COSY NMR spectrum it was possible to differentiate two spin systems attributable to two fragments of butyl and *n*-pentyl moieties (Fig. 2). A second correlation series were also observed between two protons of a methylene group at δ 2.47 (H-4´ax) and δ 2.61 (H-4 ec) with the signals at δ 3.99 (H-5) and δ 5.89 (H-2), indicating the existence in the molecule of a –CH=C–CH_2_–CH–(O)-grouping. The assignment of their NMR spectrum and location of the butyl and *n*-pentyl fragments were confirmed in the HMBC experiment with the correlations between H-2 with C-4/C-6/C-1´, H-4 with C-2/C-3/C-5/C-6/C-1´, H-5 with C-4/C-6/C-1´´, H-1´ with C-2/C-3/C-4/C-2´/C-3´ and H-1´´with C-1/C-6/C-2´´(Fig. 2). Correlations of methylene H-2’ (δ 1.45) with C-1´/C-3´/C-4´ and methyl groups, H-5´ (δ 0.88) with C-3´/C-4´ and H-4´´ (δ 0.85) with C-2´´/C-3´´ were also observed.

The molecular formula and the correlations in HMBC and ^1^H NMR-2D-COSY experiments suggested that **1** contained a 6-butyl-3-pentylcyclohexane skeleton. The relative stereochemistry of the hydroxyl groups at C-5 and C-6 was assigned based on NOESY studies. Thus, irradiation of the signal of H-4β produced NOE enhancements of the signals corresponding to H-1´´ indicating that they have an axial configuration in the β-face of the molecule, whilst irradiation on H-4α enhanced H-5 and showed that these hydrogens were located in the α-face. The β relative stereochemistry for the hydroxyl group at C-5 was also supported by the large coupling constant between H-5 and H-4β [J_H-4β-H-5_ (10 Hz)], indicative of a *trans* configuration of these protons in the ring. The relative position of the butyl moiety was also confirmed by NOE effects between protons H-4β and H-1´´, H-2 and H-1´. All these data allowed us to assign the structure 6-butyl-5,6-dihydroxy-3-pentylcyclohex-2-en-1-one (**1**) to this new compound and we have named stempholone A. The absolute configuration of **1** was not determined. This compound was first described in a patent application of our research group^11^.

Compound **2** was an amorphous brown solid. The ^1^H and ^13^C NMR (Table 1) displayed signals very similar to stempholone A, and its high resolution mass spectrum exhibited an ion peak at *m*/*z* 270.1833 (C_15_H_26_O_4_, calcd. 270.1831), which indicates the introduction of a new oxygen atom into the molecule. The main significant difference observed in ^1^H-NMR with respect to **1** was the absence of one aliphatic methyl group and the presence of a doublet at δ 1.20, due to a methyl group (3H, *d*, J = 6.0 Hz), coupled with a new signal at δ 3.82 (1H, *q*, *J* = 6.0 Hz) of a proton geminal to hydroxyl group. The corresponding carbons were observed in the ^13^C NMR spectrum at δ 23.8 (C-5´) and 67.7 (C-4').

These data suggested that these two compounds differ in the presence of one additional hydroxyl group in **2**, which must be located at C-3´´ or C-4´. The correlation observed in the two-dimensional HMBC experiment between of methyl group at δ 1.20 (3H, *d*, H-5´) and the hydroxy methine at δ 67.7 (C-4´) confirms the position of the hydroxyl group at C-4´´ On the other hand, the NOE effect between H-1´´ and H-4β as well as coupling constants of H-5 and H-4 β -allowed determinate the relative stereochemistry at C-5 and C-6. These observations, together with a careful analysis of the COSY and HMBC experiments, led to the identification of compound **2** as 6-butyl-5,6-dihydroxy-3-(4-hydroxypentyl)cyclohex-2-en-1-one. A detailed analysis of the ^13^C NMR showed double signals for C-2´, C-3´, C-4´ and C-5´, indicating that compound **2** was a mixture of two epimers at C-4´ (ratio 1:1). This compound was first described in a patent application of our research group^13^ and also from a fungal strain isolated from the gut of a marine isopod (*Ligia oceanica*)^14^.

Compound **3** was isolated as a white solid. The molecular formula was determined to be C_15_H_24_O_2_ from the molecular ion peak at *m*/*z* 236.1772 (Calcd.236.1776) in the HR-EIMS. The ^1^H NMR of compound **3** revealed signals representative of two *n*-alkyl chains (butyl and *n*-pentyl moieties), a singlet signal integrated for two protons at δ 6.22 (H-4, H-6) and two hydroxyl groups at δ 4.62 (2H, *s*, OH). The chemical shift and multiplicity suggested the presence of a 1,3-dihydroxy-2,5-tetrasubstituted phenyl ring. This proposal was further supported by detailed analysis of the ^13^C-NMR and 2D-NMR data. Spectroscopic data of **3**^15,16^ were identical to those previously reported for stemphol.

Compounds **1**–**3** were further tested against the identified targets according to the bioguided fractionation data (Tables S1 and S2 in Supplementary Materials). The antifungal effects are shown in Table 2. Compound **1** was moderately active on *B. cinerea* (EC_50_ value of 0.43 mg/mL), **2** was active on *F. solani* (EC_50_ value of 0.21 mg/mL) with lower potency than the extract, and stemphol (**3**) was antifungal to *F. moniliforme* (EC_50_ value of 0.02 mg/mL). These effective doses were in the range of the positive control thymol for stemphol (**3**) on *F. moniliforme* (EC_50_ of 0.053 for thymol) and were about 10 times less effective against the other fungal targets (EC_50_ of 0.049 and 0.053 mg/mL on *B. cinerea,* and *F. solani*).

**Table 2:** Antifungal effects on *Fusarium moniliforme, F. solani* and *Botrytis cinerea* mycelial growth of extract (EtOAc) and compounds (**1**–**3**) of *Stemphylium solani.*

Sample	EC_50_ mg/mL (95% CL)^a^		
	*F. moniliforme*	*F. solani*	*B. cinerea*
Extract	0.19 (0.19–0.19)	0.08 (0.079–0.08)	0.31 (0.30–0.31)
**1**	Nt	Nt	0.43 (0.42–0.43)
**2**	> 0.5	0.21 (0.21–0.22)	> 0.5
**3**	0.02 (0.020–0.021)	> 0.5	> 0.5

*Nt* not tested.

^a^Efficient dose to give 50% inhibition and 95% confidence limits (CL).

When tested on insect pests (Table 3), compounds **1**–**3** were all active on the aphid *M. persicae*, with **2** and **3** (EC_50_ values of 0.2 and 0.5 gμ/cm^2^ respectively) being more effective than **1** (EC_50_ value of 1.5 gμ/cm^2^), the extract (EC_50_ value of 6.0 gμ/cm^2^) or the positive control thymol (EC_50_ value of 7.6 gμ/cm^2^). *M. javanica* was more affected by compound **1** than by the extract, with a minimum lethal dose (MLD for 86% mortality) of 0.5 mg/mL (MLD 90% for the positive control thymol is 0.22 mg/mL).

**Table 3:** Biocidal effects of *Stemphylium solani* extract (EtOAc) and compounds (**1**–**3**) against insect pests (antifeedant effects on *Spodoptera littoralis, Myzus persicae, Rhoplalosiphim padi*) and the nematode *Meloidogyne javanica* (% mortality of juveniles J2).

Target	Effect	Treatment	Activity (%)	EC_50_^c^/MLD^d^
*S. littoralis*	%FI^a^	Extract	46.5 ± 11.8	> 100
		**1**	41.8 ± 7.9	> 50
		**2**	52.9 ± 5.9	> 50
		**3**	60.4 ± 8.7	> 50
*M. persicae*	%SI^a^	Extract	99.5 ± 0.5	6.0 (3.7–9.8)
		**1**	81.4 ± 5.6	1.5 (0.6–3.6)
		**2**	83.9 ± 6.9	0.2 (0.03–1.5)
		**3**	85.3 ± 5.4	0.5 (0.1–3.0)
*R. padi*	%SI^a^	Extract	40.0 ± 6.8	> 100
		**1**	nt	> 50
		**2**	45.1 ± 9.1	> 50
		**3**	72.4 ± 5.6	> 25
*javanica*	% Mortality^b^	Extract	68.21 ± 2.05	> 1.0
		**1**	83.05 ± 3.15	0.5
		**2**	1.24 ± 0.90	> 1.0
		**3**	1.24 ± 0.68	> 1.0

^a^Doses tested of 100μ g/cm^2^ for the extract and 50.0–12.2 gμ/cm^2^ for the compounds on insects.

^b^Doses tested of 1 gμ/mL for the extract and 0.5–0.25 mg/mL for the compounds on *M. javanica.*

^c^Efficient dose (μg/cm^2^) to give 50% feeding inhibition (Feeding/Settling) and 95% confidence limits (lower, upper).

^d^Minimum Lethal Dose (mg/mL) to give 80% mortality of *M. javanica.*

**Figure 3:**
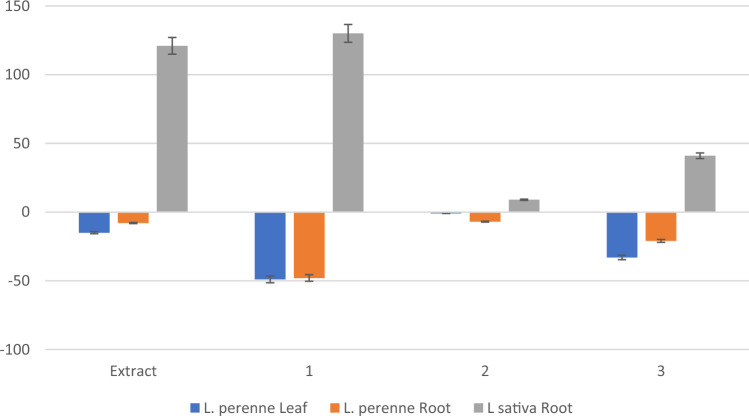
Phytotoxic effects of extract (EtOAc) and compounds (**1**–**3**) of *Stemphylium solani* on *Lolium perenne* and *Lactuca sativa* leaf and root growth (doses tested of 0.4 and 0.1 mg/mL for the extract and **1**–**3** respectively).

The phytotoxicity tests on *L. perenne* and *L. sativa* showed that none of the treatments affect germination. Figure 3 shows the effects of the extracts and **1**–**3** on root and leaf growth. Compound **1** increased *L. sativa* root growth (130% increase) similarly to the extract (121%). Compound **1** reduced *L. perenne* growth (leaf and root: 48–49% decrease), while compound **3** showed only moderate (< 50%) effects (*L. perenne* leaf and root growth: 21–33% decrease, *L. sativa* root growth: 41% increase).

## Discussion

In this study, the endophytic fungi Aa22, isolated from leaves of *Artemisia absinthium,* has been identified as *Stemphylum solani*. The genus *Stemphylium* (Pleosporaceae, Pleosporales) contains 96 species and includes plant pathogenic, endophytic, and saprophytic species with worldwide distributions^17^. *Stemphylium* species have been isolated as endophytes from marine and terrestrial sources^18–21^. Specifically, *S. solani* has been isolated as a plant endophyte from *Mentha pulegium*^22,23^. *Arabidopsis thaliana*^24^, *Vitis vinifera*^25^, the endemic Australian species *Eremophilia longifolia*^26^ and *Sonchus asper*^27^. This is the first report on an endophytic *S. solani* from *A. absinthium.*

The bio-guided chemical study of the active Aa22 EtOAc extract gave the following compounds: stempholone A (**1**) and stempholone B (**2**). Stempholones A and B were first described in a patent application of our research group^13^ and later, **2** was isolated from a marine isopod fungal strain^12^. Compound **3,** identified as stemphol^15,16^, was isolated for the first time as a phytotoxin from *Stemphylium majusculum*^15^.

Marine-derived endophytic *Stemphylium* species produce bianthraquinones, anthraquinones, meroterpenoids, dialkylresorcin derivatives including stemphol^28–31^. Plant endophytic *Stemphylium* sp. have been reported to produce compounds such as stemphol sulfates, stemphol and anthraquinone derivatives from mangrove endophytic isolates^16^, taxol, from *S. sedicola* isolated from *Taxus baccata*^20,23^, Δ8-pregnene steroids from *Polialthya laui* isolates^32^, altersolanols, alterporriols and anthracene derivatives from *S. globuliferum* isolated from *Mentha pulegium*^22,23,32^. Most of these natural products have promising biological and pharmacological activities, such as cytotoxic and antimicrobial^18–20,22,23,33–36^, including stempholone B (**2**)^14^.

The crop protection effects of *Stemphylium* metabolites are not known (only in patent^13^). Recently, a direct plant protective effect has been reported for a fungal isolate of *Stemphylium majusculum,* from cotton plants, when inoculated in soybean by reducing the leaf area consumed by caterpillars (*Trichoplusia ni* and *Chrysodeixis includens*) and the number of cysts produced by nematodes (*Heterodera glycines*)^37^. However, this is the first report on the antifungal, insect antifeedant or nematicidal effect for an *S. solani* extract and compounds **1**–**3** (reported in patent^13^).

Overall, the extract was an effective antifungal against *Fusarium* sp. probably due to is content in stemphol **3**. Stemphol has been reported as an antibacterial agent^16^, but this is the first report on its antifungal effects on plant pathogens. The aphid antifeedant activity of the extract against *M. persicae* can be explained by the active compounds **1**–**3**, while the nematicidal activity of the extract can be explained by **1**.

The monocotyledonous species *L. perenne* was negatively affected by **1** and only moderately by **3**, while the extract and **1** increased the root growth of the dicotyledonous plant *L. sativa*. Therefore, **1**, with a hydroxylation in C6, showed a similar, but stronger, phytotoxic pattern to **3**, while a further hydroxylation (C-4´), such as compound **2**, eliminated this effect. Stemphol (**3**) has been reported as being cytotoxic to isolated cells of oilseed rape and chickpea^33^, but this is the first report on the phytotoxic effects of stempholones **1** and **2**.

It is interesting to note that the related compounds stempholone **1** and** 2**, gave different bioactivity profiles depending on the target: **1** was antifungal to *B. cinerea*, nematicidal, phytotoxic to *L. perenne* and stimulated the growth of *L. sativa* root, while **2** was the most antifeedant to *M. persicae.* Therefore, a fermentation optimization is needed to increase and/or modulate the production of **1**–**3** for the production of customized bioactive Aa22 extracts.

## Conclusions

In this study the endophytic fungi Aa22, isolated from leaves of *Artemisia absinthium*, has been identified as *Stemphylum solani*. A bioactive EtOAc extract from the rice fermentation of this strain was chromatographed to afford the metabolites stempholone Overall, the extract was an effective antifungal against *Fusarium* sp probably due to is content in stemphol **3**. The aphid antifeedant activity of the extract against *M. persicae* can be explained by the active compounds **1**–**3**, while the nematicidal activity of the extract can be explained by **1**. The monocotyledonous species *L. perenne* was negatively affected by **1,** while the extract and **1** increased the root growth of the dicotyledonous *L. sativa.* Compound **3** had low phytotoxic effects on* L. prenne.*

Therefore, the endophytic strain Aa22 (*S. solani*) has the potential to develop antifungal and aphid antifeedants with low phytotoxic effects to dicotyledonous plants. Further optimization of the fermentation process is needed to increase the active components and the bioactivity of these fungal extracts and to reduce the fermentation time.

## Materials and methods

### General experimental procedure

Optical rotations were determined at room temperature on a Perkin Elmer 343 polarimeter (Perkin Elmer, Waltham, MA, USA). IR spectra was recorded on Bruker IFS 66/S spectrometer. NMR spectra (^1^H and ^13^C) were measured on a Bruker AMX-500 spectrometer (^1^H 500 MHz/^13^C 125 MHz) with pulsed field gradient using the solvent as internal standard (CDCl_3_, at δ_H_ 7.26 and δ_C_ 77.0). The programs used in two-dimensional (2D) NMR experiments (HMBC, HSQC, COSY and NOESY) were as per the manufacturer’s software (Bruker Corporation, Billerica, MA, USA). High-resolution MS spectra (HRESIMS) were recorded on a Micromass Autospec instrument at 70 eV. Column chromatography (CC) and Vacuum Liquid Chromatography (VLC) were performed with Silica gel 0.025–0.04 and 0.040–0.015 mm (Macherey–Nagel GmbH&Co.KG, Düren, Germany). Sephadex LH-20 (Sigma-Aldrich, St. Lo Sigma-Aldrich, St. Louis, MO, USA) was used for size exclusion column chromatography (CC). The eluted fractions from column chromatographic separations were analyzed by TLC using precoated silica gel 60 F254 plates (Merck). Visualization of spots was done under a UV lamp (254 and 365 nm) and by spaying the plates with with H_2_SO_4_ (5%) or vanillin (50 mg/mL in a 5% H_2_SO_4_ solution in EtOH).

### Isolation and characterization of fungal endophyte

The endophytic fungi were isolated from a registered variety (Candial) of *Artemisia absinthium,* developed by the research group and cultivated in Ejea de los Caballeros, Spain (42.1257° N, 1.1365° W) as described^11,12^. Plant fresh material (aerial parts) was collected in 2013 from a 4 years old plant (from block 2 of the experimental field) were surface sterilized with ethanol (75% for 2 min) followed by sodium hypochlorite (3% for 3 min), followed by washing with sterile distilled water. Surface-disinfected samples (leaves and stem) were cut into small segments (0.5 cm) and the tissue segments were placed on Petri dishes coated with two culture media: Potato Dextrose Agar (PDA) with chloramphenicol and Yeast Mannitol Agar (YMA) with tetracycline. Antibiotics (50 mg/L) were added to inhibit bacterial growth. Petri dishes were incubated at 27 ºC in darkness for 3–15 days in a growth chamber. Fungal colonies growing in YMA dishes were transferred in to new plate to obtain pure strain and further identification.

The endophyte fungi isolated were fermented on rice for 21 days and screened for their activity against plant pathogens, insects and root-knot nematodes as described below and the isolate Aa22 obtained from the leaves was selected for this study. The molecular identification of Aa22 was carried out by 16S rRNA gene sequencing based on the amplification (PCR) and sequencing of the ribosomal ITS region of the rDNA^3^. Briefly, genomic DNA (100–200 ng) was amplified (PTC-200 Thermal Cycler, MJ Research, San Diego, CA, USA), 25 µL final volume with AmpONE Taq DNA polymerase PCR kit (GeneAll, Seoul, Korea) with 35 cycles (95 °C, 1 min; 50 °C, 20 s; 72 °C, 1.5 min) after an initial denaturation (95 °C, 2 min) followed by a final extension (72 °C, 7 min). Amplicons were checked by agarose gel (1%) electrophoresis, purified using the EXO-SAP-IT kit (Affimetrix-USB; Thermo Fisher Scientific, Waltham, MS, USA), and sequenced on an AB 3500 Genetic Analyzer (Thermo Fisher Scientific, Waltham, MS, USA) at the University of La Laguna (La Laguna, Spain) genomic service. The obtained ITS1-5.8S-ITS2 sequence data (Sequence data in supplementary material) was compared with these published in the NCBI (National Center for Biotechnology Information, https://www.ncbi.nlm.nih.gov/) database using Basic Local Alignment Search Tool (nBLAST), and matched accession numbers JF913269.1 and AF203451.1 (100%) from *Stempilium solani* (Genbank: https://www.ncbi.nlm.nih.gov/nuccore/JF913269.1; https://www.ncbi.nlm.nih.gov/nucleotide/AF203451.1?report=genbank&log$=nuclalign&blast_rank=4&RID=3R6UH7MT013). A sample of this isolate AA22 has been deposited in “Colección Española de Cultivos Tipo” (CECT) with number 20941 (Valencia, Spain).

### Fungal fermentation

The fungus was cultivated in potato dextrose agar (PDA) medium at 25 °C for two weeks to prepare the seed culture. Six (2.5 cm × 2.5 cm) of fresh mycelium were inoculated into each twelve Erlenmeyer flasks (250 ml) containing 100 g of rice and 30 ml of distilled water. The fungal was grown on solid rice medium in darkness at 25 °C for three weeks^3^.

### Extraction and isolation

After 21 days of incubation, the static fermentation on rice of *S. solani* was extracted four times with ethyl acetate (EtOAc) at room temperature. Each flask was filled with EtOAc (300 mL) and keeping in maceration for 24 h. The supernatant was vacuum filtered using a Büchner funnel and the organic solvent was evaporated until dryness under reduced pressure to afford 4.58 g of crude extract^3^. The methanol-soluble fraction (4.09 g) of the crude extract was fractioned by vacuum-liquid chromatography column (VLC) on silica gel 60 (0.3 kg) using hexane/ethyl acetate mixtures of increasing polarity, acetone and methanol/water mixtures to yield eight fractions: H1-H8. Fraction H6 (acetone, 0.452 g) was submitted to CC using hexane/ ethyl acetate mixtures of increasing polarity on silica gel. Less polar fractions eluted with hexane/EtOAc (90:20–90:30) yielded **1** (19 mg). Further purification of the one of the most polar fractions (hexane/EtOAc 50:50) on Sephadex LH-20 using hexane/chloroform /methanol (2:1:1) yielded **2** (11 mg).

Fraction H2 (hexane/EtOAc, 9:1, 0.575 g) was subjected to (CC) on silica gel using mixtures of hexane/acetone afforded six subfractions (H2A-H2F). Fraction H2E (0.053 g) was further chromatographed on silica gel CC using mixtures of hexane/chloroform as mobile phase to yield the pure compound **3** (21 mg). Fraction H3 (hexane/EtOAc, 4:1, 0.107 g) was subjected to CC on Sephadex LH-20 using hexane/chloroform /methanol (2:1:1) to afford again **3** (74 mg).

*Stempholone A* (**1**): [α]_D_ + 4.4 (c 0.08, CHCl_3_); IR (film) ν_max_ 3443, 2957, 2930, 2861, 1673, 1650, 1626, 1467, 1378, 1257, 1142, 1075 cm^-1^; ^1^H NMR data (CDCl_3_, 500 MHz), see Table 1; ^13^C NMR data (CDCl_3_, 125 MHz), see Table 1; HREIMS *m/z* 254.1888 [M]^+^ (calcd for C_15_H_26_O_3_, 254.1882); EIMS 70 eV m*/z* (rel. int.): 254 [M]^+^ (5), 198 (35), 179 (8), 169 (33), 151 (21), 139 (100), 124 (22), 116 (58), 95 (20), 85 (54), 74 (55).

*Stempholone B* (**2**): [α]_D_ + 12.5 (c 0.056, CHCl_3_); IR (film) ν_max_ 3418, 295, 2931, 2872, 1673, 1667,1651,1626, 1433, 1377, 1260, 1138, 1076 cm^-1^; ^1^H NMR data (CDCl_3_, 500 MHz), see Table 1; ^13^C NMR data (CDCl_3_, 125 MHz), see Table 1; HREIMS *m/z* 270.1833 [M]^+^ (calcd for C_15_H_26_O_4_, 270.1831); EIMS 70 eV m*/z* (rel. int.): 270 [M^+^] (7), 252 (14), 197 (35), 179 (12), 167 (33), 155 (17),149 (26), 137 (21), 125 (10), 116 (100), 109(28), 95 (54), 85 (85), 74 (96).

*Stemphol* (**3**): IR (film) ν_max_ 3300, 2850, 1630, 1588, 1430, 1270 cm^-1^; ^1^H NMR (CDCl_3_, 500 MHz) δ: 0.88 (3H, t, *J* = 7.0 Hz, H-5´´), 0.93 (3H, t, *J* = 7.0 Hz, H-4´), 1.31 (2H, m, H-4´´), 1.40 (2H, m, H-3´), 1.51 (6H, m, H-2´, H-2´´and H-3´´), 2.44 (2H, t, *J* = 8.0 Hz, H-1´´), 2.58 (2H, t, *J* = 8.0 Hz, H-1´), 6.26 (2H, s, H-4 and H-6); ^13^C NMR (CDCl_3_, 125 MHz), δ: 154.4 (C-1), 112.5 (C-2), 154.4 (C-3), 108.1(C-4), 142.2 (C-5), 108.1 (C-6), 22.8 (C-1´), 31.4 (C-2´´), 22.7 (C-3´), 13.9 (C-4´), 35.5 (C-1´´), 30.7 (C-2´´), 31.5 (C-3´´), 22.7 (C-4´´), 13.9 (C-5´´); HREIMS *m/z* 236.1771 [M]^+^ (calcd for C_15_H_24_O_2_, 236.1776); EIMS 70 eV m*/z* (rel. int.): 236 [M^+^] (16), 194 (16), 193 (100), 180 (45), 137 (7), 123 (11), 91 (3), 77 (4).

^1^H-NMR, ^13^C-NMR, COSY, HSQC, HMBC, NOESY and HREIMS spectra can be found in Supplementary Materials for compounds **1** and **2** (Figs. S1–S16).

### Antifungal bioassay

*Fusarium moniliforme* (Sheldon) [CECT2152] and *F. solani* (Mart) [CECT2199] were provided by CECT. *Botrytis cinerea Pers*. [strain B05.10] was provided by the Department of Biochemistry from University La Laguna (Spain).

Antifungal activity was analyzed as mycelial growth inhibition by a modified agar-dilution method, which includes the addition of 0.05 mg/mL of methyltetrazolium salts (MTT) to improve visualization of the mycelium^12^. Test samples were dissolved in EtOH and solutions were added into PDB culture medium (5 mL) at a range of concentrations (extract at 1–0.01 mg/mL, compounds at 0.5–0.01 mg/mL). The final EtOH concentration was identical in both the control (only solvent) and treated cultures. The medium was poured into 9 cm diameter sterile Petri dishes and a fungal mycelium disk was placed on the solidified agar medium. Eight replicates were used for each concentration. After incubation in darkness at 27 °C for 48 h, fungal colonies were digitalized and measured with the application ImageJ (http://imagej.nih.gov/ij/, accessed on 23 November 2023).

Percent inhibition (%I) was calculated as: %I = (C − T/C) × 100, where *C* is the diameter of the control colonies and *T* is the diameter of the test colonies. Data were analyzed with Statgraphics statistical analysis software (Centurion XVIII) and EC_50_ values (effective dose to obtain 50% of inhibition) were determined by means of a regression curve of mycelial growth inhibition versus log dose. Thymol (Sigma Aldrich) was used as a positive control with effective doses (EC_50_) of 0.049, 0.050 and 0.059 mg/mL on *B. cinerea*, *F. solani* and *F. moniliforme,* respectively.

### Antifeedant activity

*Spodoptera littoralis, Myzus persicae* and *Rhopalosiphum padi* colonies were reared on artificial diet, bell pepper (*Capsicum annuum*) and barley (*Hordeum vulgare*) plants, respectively at ICA-CSIC. The insect colonies and host plants were maintained at 22 ± 1 ºC, > 70% relative humidity with a photoperiod of 16:8 h (L:D) in a growth chamber.

Bioassays were conducted with 1.0 cm^2^ leaf disks / fragments of *C. annuum* (*M. persicae, S. littoralis*) or *H. vulgare* (*R. padi*). The tests (10 μL of the solution) were applied at initial doses of 10 or 5 mg/mL (extract or product) to the upper surface of the leaf fragments^38^. Apterous aphid adults (24–48 h old) were placed in 20 (2 × 2 cm) ventilated plastic boxes (10 per box) and kept in a growth chamber for 24 h. Two sixth-instar *S. littoralis* larvae (> 24 h after moulting) were placed in 6 Petri dishes with 2 leaf disks (treatment disk with the test solution and control disk with solvent) and allowed to feed until 75% larval consumption of any pair of these disks. These experiments were repeated twice (SE < 10%).

Settling (S, aphid species) was measured by counting the number of insects on each leaf fragment, and feeding (F, *S. littoralis*) was measured calculating the disk surface consumption (disks digitalized with https://imagej.nih.gov/ij/, accessed on 23 November 2023). The feeding or settling inhibition (%FI or %SI) was calculated as [1—(T/C) × 100], where T and C represent treated and control leaf fragments, respectively. The effects (%SI/%FI) were analyzed by the non-parametric Wilcoxon Signed-Rank Test. Extracts and compounds with an effect ≥ 70% were tested in dose–response experiments (3–5 serial dilutions) to calculate their EC_50_ (the effective dose causing a 50% settling/feeding reduction) with linear regression models (%FI/SI on Log-dose). The positive control for *M. persicae* was Thymol (Sigma Aldrich) with an EC_50_ of 7.6 gμ/cm^2^.

### Nematicidal activity

A field-selected *M. javanic*a population from Barcelona, Spain, maintained on tomato plants (*Solanum lycopersicum* L. var. *Marmande*) cultivated in pot cultures and kept in plant growth chambers (25 ± 1 °C and > 70% relative humidity) has been used. Two months after seedling inoculation, second-stage juveniles (J2) were obtained by incubating egg masses, collected from the infected roots, in water at 25 °C for 24 h.

The tests were carried out in 96-well plates (BD Falcon, San Jose, CA, USA) as described^39^. Each well contained 100 J2 juveniles, 95 mL of water and 5 μL of the the test solution (10 mg/mL in 0.2% Tween-20 in DMSO), resulting in an initial concentration of 1 or 0.5 mg/mL (extract or compound). Solvent (0.2% Tween-20 in DMSO) was used for the negative control and all experiments were replicated 4 times.

The results are presented as percentage of dead J2 corrected according to Scheider-Orelli's formula. The dose–response (5–8 concentrations) mortality data is expressed as Minimum Lethal Dose (MLD, mg/mL) to give 80% J2 mortality. Thymol (Sigma Aldrich) was used as a positive control, with an MLD of 0.22 mg/mL.

### Phytotoxic activity

For the phytotoxicity tests, ryegrass (*Lolium perenne*) and tomato (*S. lycopersicum*) seeds (40 seeds per test) placed in 12-well microplates (with three replicas) were used^40^. The extracts or products dissolved in solvent (acetone or methanol) were applied on paper disks (2 cm diameter, 200 or 100 gμ/disk of extract or compound respectively plus 500μL distilled water) were tested at 0.4 or 0.1 mg/mL respectively (final concentration per well). Solvent was used for negative control and the positive control was Juglone (Sigma) (0.1 mg/mL, 100% germination inhibition).

The germination was checked for seven days, and the leaf (for ryegrass) or root (for both species) length was measured on a total of 25 randomly selected digitalized seedlings (https://imagej.nih.gov/ij/, accessed on 23 November 2023). A non-parametric analysis of variance (ANOVA) was performed on the root/leaf length data. The data is expressed as relative percent respect to the negative control (control = 0% for each parameter measured).

### Plant material statement

Experimental research and field studies on plants (cultivated and wild), including the germplasm collection used in this work comply with institutional, national, and international guidelines and legislation.

### Supplementary Information

Supplementary Information.

## Data Availability

The obtained ITS1-5.8S-ITS2 sequence data was compared with these published in the NCBI (National Center for Biotechnology Information, https://www.ncbi.nlm.nih.gov/) database using Basic Local Alignment Search Tool (nBLAST) (accession number in Genbank: https://www.ncbi.nlm.nih.gov/nuccore/JF913269.1). The data that support the findings of this study are available from the corresponding author upon reasonable request.
