# Supplementary material for: Antifeedant, antifungal and nematicidal compounds from the endophyte Stemphylium solani isolated from wormwood
Source: Sci Rep. 2024 Jun 12;14:13500. doi: 10.1038/s41598-024-64467-w (PMC11169264; doi:10.1038/s41598-024-64467-w)
Supplement: Supplementary file 1 — Supplementary Information. [file 41598_2024_64467_MOESM1_ESM.pdf]

Antifeedant, antifungal and nematicidal compounds from the endophyte *Stemphylium solani* isolated from wormwood

Carmen E. Díaz[1], María Fe Andrés[2], Rodney Lacret[2], Raimundo Cabrera[3], Cristina Gimenez[3], Nutan Kaushik[4] and Azucena González-Coloma*[2]

[1]*Instituto de Productos Naturales y Agrobiología, Consejo Superior de Investigaciones Científicas. Avda. Astrofísico F. Sánchez 3, 38206 La Laguna, Tenerife, Spain.*

[2]*Instituto de Ciencias Agrarias, Consejo Superior de Investigaciones Científicas. Serrano 115, 28006 Madrid*

[3]*Departamento de Botánica, Ecología y Fisiología, Facultad de Ciencias, Sección Biología, Universidad de La Laguna, 38206 La Laguna, Tenerife, Spain*

[4]*The Amity Food and Agriculture Foundation, Amity University, Uttar Pradesh, Noida, 201313, India*

____

* Corresponding author:

Dr. Azucena González-Coloma.

azu@ica.csic.es

List of contents:

Sequence data of Aa22 *Stemphylium solani*

Table S1. Bioactivity of Aa22 fractions

Table S2. Phytotoxic effects of Aa22 fractions

Figure S1. ^{1}H-NMR spectrum of compound **1** ($CDCl_3$, 500 MHz)

Figure S2. ^{13}C-NMR spectrum of compound **1** ($CDCl_3$, 125 MHz)

Figure S3. HSQC spectrum of compound **1** ($CDCl_3$, 500 MHz)

Figure S4. COSY spectrum of compound **1** (CDCl3, 500 MHz)

Figure S5. HBMC spectrum of compound **1** (CDCl3, 500 MHz)

Figure S6. NOESY 1D spectrum of compound **1** (CDCl3, 500 MHz)

Figure S7. NOESY 1D spectrum of compound **1** (CDCl3, 500 MHz)

Figure S8. NOESY 2D spectrum of compound **1** (CDCl3, 500 MHz)

Figure S9. HREIMS spectrum of compound **1**.

Figure S10. ^{1}H-NMR spectrum of compound **2** ($CDCl_3$, 500 MHz)

Figure S11. ^{13}C-NMR spectrum of compound **2** ($CDCl_3$, 125 MHz)

Figure S12. HSQC spectrum of compound **2** ($CDCl_3$, 500 MHz)

Figure S13. COSY spectrum of compound **2** ($CDCl_3$, 500 MHz)

Figure S14. HMBC spectrum of compound **2** ($CDCl_3$, 500 MHz)

Figure S15. NOESY 2D spectrum of compound **1** (CDCl3, 500 MHz)

Figure S16. HREIMS spectrum of compound **1**.

Sequence data of fungus *Stemphylium solani*

>Aa22

TCTCCGTAGGTGAACCTGCGGAGGGATCATTACACAATATGAAAGCGGGCTGGGACCTTACTTCGGTGAG

GGCTCCAGCTTGTCTGAATTATTCACCCATGTCTTTTGCGCACTTCTTGTTTCCTGGGCGGGTTCGCCCG

CCACCAGGACCAAACCATAAACCTTTTTGTAATTGCAATCAGCGTCAGTAAACAATGTAATTATTACAAC

TTTCAACAACGGATCTCTTGGTTCTGGCATCGATGAAGAACGCAGCGAAATGCGATACGTAGTGTGAATT

GCAGAATTCAGTGAATCATCGAATCTTTGAACGCACATTGCGCCCTTTGGTATTCCAAAGGGCATGCCTG

TTCGAGCGTCATTTGTACCCTCAAGCTTTGCTTGGTGTTGGGCGTCTTGTCTCTCACGAGACTCGCCTTA

AAATCATTGGCAGCCGACCTACTGGTTTCGGAGCGCAGCACAATTCTTGCACTTTGAATCAGCCTTGGTT

GAGCATCCATCAAGACCCTATTTTTTTTAACTTTTGACCTCGGATCAGGTAGGGATACCCGCTGAACTTA

AGCATATCAATAAGCGGAGG

Table S1. Biocidal effects of *Stemphylium solani* fractions against *Fusarium moniliforme, F. solani* and *Botrytis cinerea* mycelial growth, insect pests (antifeedant effects on *Spodoptera littoralis, Myzus persicae, Rhoplalosiphim padi*) and the nematode *Meloidogyne javanica* (% mortality of juveniles J2).

	**Mycelial growth inhibition (%)**			**%SI**	**% Mortality**
	**(0.5 mg/mL)**			**100μg/cm^2**	**(1 mg/mL)**
	Fusarium moniliforme	***F. solani***	***Botrytis cinerea***	***Myzus persicae***	***Meloidogyne javanica***
H1	3.52±0.78	1.12±1.62	9.61±3.66	50.29 ± 10.21	0.41 ± 1.60
H2	62.79±1.72	46.79±6.89	9.44±3.77	92.76 ± 2.59	7.46 ± 1.79
H3	66.26±1.69	52.55±2.9	54.82±1.97	91.51 ± 3.28	0.00 ± 0.00
H4	0	15.26±2.78	1.03±1.13	63.91 ± 4.06	0.00 ± 0.00
H5	12.87±2.86	15.32±3.00	16.03±2.30	47.54 ± 10.06	10.39 ± 1.87
H6	22.93±3.48	66.45±3.54	62.83±2.72	94.88 ± 6.77	70.14 ± 3.23
H7	0	4.62±1.93[a]	0.24±2.67	63.18 ± 2.15	0.98 ± 1.42
H8	0	4.58±2.43	3.87±2.81	62.04 ± 5.65	1.80 ± 0.65

Table S2. Phytotoxic effects of Aa22 fractions (dose of 0.4 mg/mL). Data is expressed as percent of the negative control

Fraction	*Lolium perenne*			*Lactuca sativa*	
	**Germination (%C)**	**Growth (%C)**		**Germination (%C)**	**Growth (%C)**
	**168h**	**Root**	**Leaf**	**72h**	**Root**
H1	86.49 ± 7.68	103.69 ± 11.26	88.93 ± 12.90	100 ± 0.00	104.77 ± 8.74
H2	100.00 ± 7.32	94.76 ± 9.89	98.61 ± 9.90	100 ± 0.00	109.26± 9.57
H3	100.00 ± 0.00	99.76 ± 5.49	96.59 ± 6.70	100 ± 0.00	133.26± 9.57
H4	100 ± 7.22	105.09 ± 11.44	81.28 ± 9.78	100 ± 0.00	103.66 ± 13.09
H5	106.25 ± 8.84	109.45 ± 13.49	103.50 ± 11.50	100 ± 0.00	107.41 ± 13.10
H6	68.75 ± 23.38	56.24 ± 8.83	58.56 ± 8.77	102.63 ± 4.08	246.57 ± 13.61
H7	88.38 ± 10.08	85.84 ± 8.32	81.77± 11.82	100 ± 0.00	147.65 ± 16.18
H8	97.30 ± 5.03	102.55 ± 9.96	80.22 ± 10.78	100 ± 0.00	104.77± 9.29

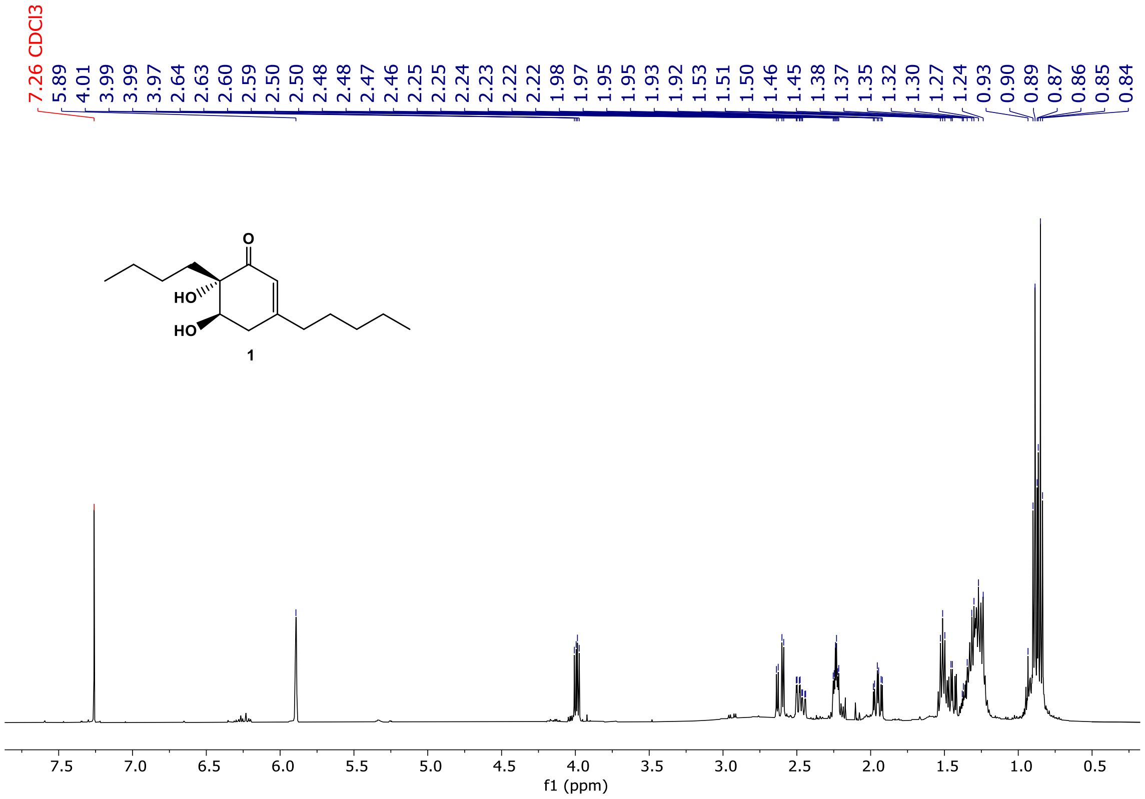

Figure S1. ^{1}H-NMR spectrum of compound **1** ($CDCl_3$, 500 MHz)

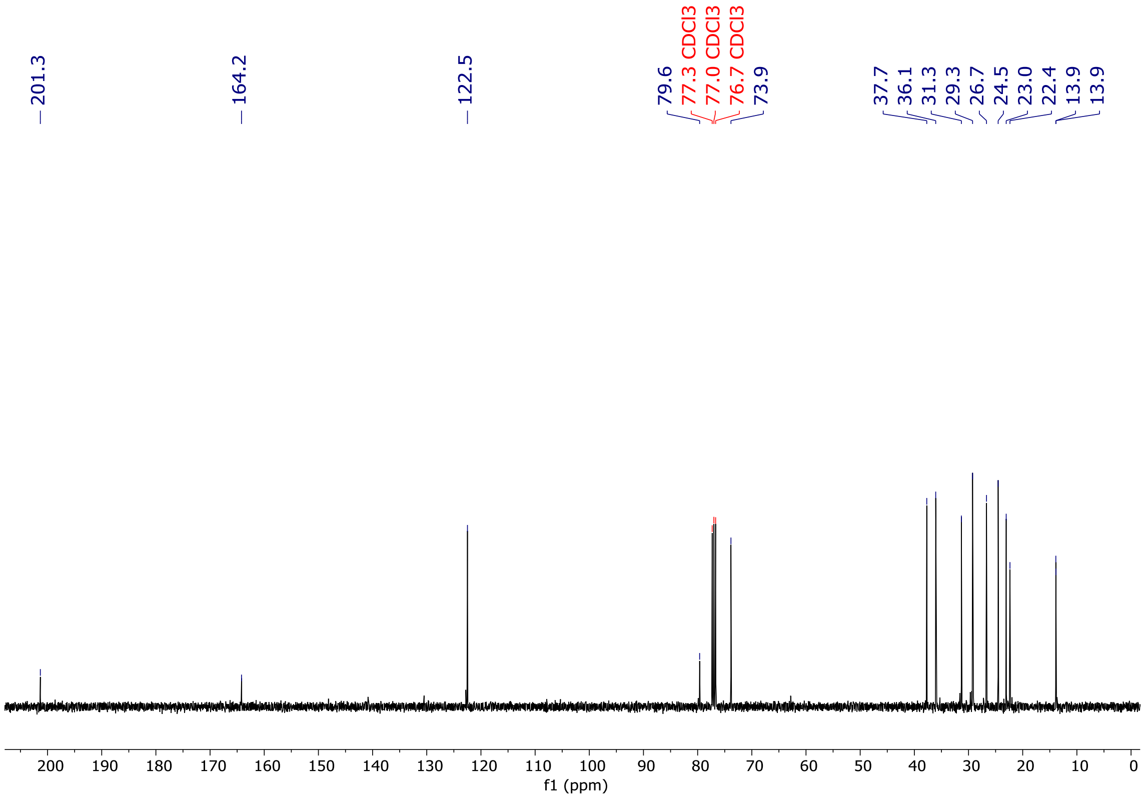

Figure S2. ^{13}C-NMR spectrum of compound **1** ($CDCl_3$, 125 MHz)

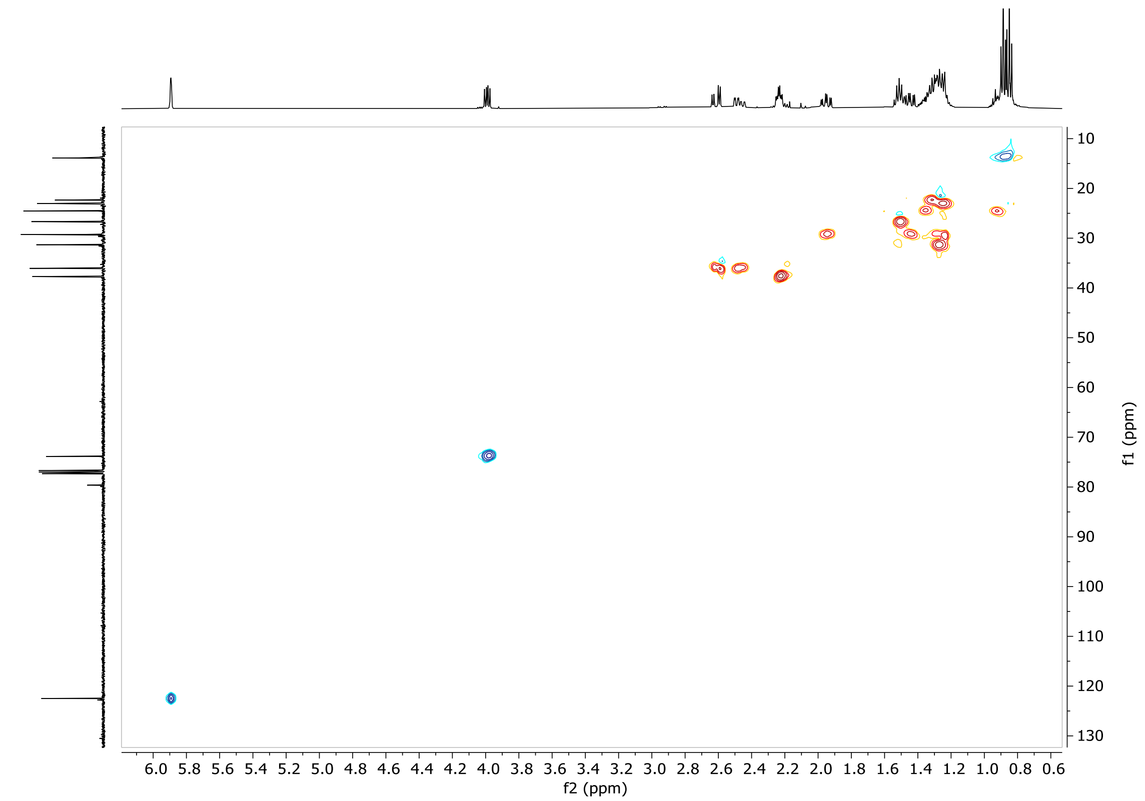

Figure S3. HSQC spectrum of compound **1** ($CDCl_3$, 500 MHz)

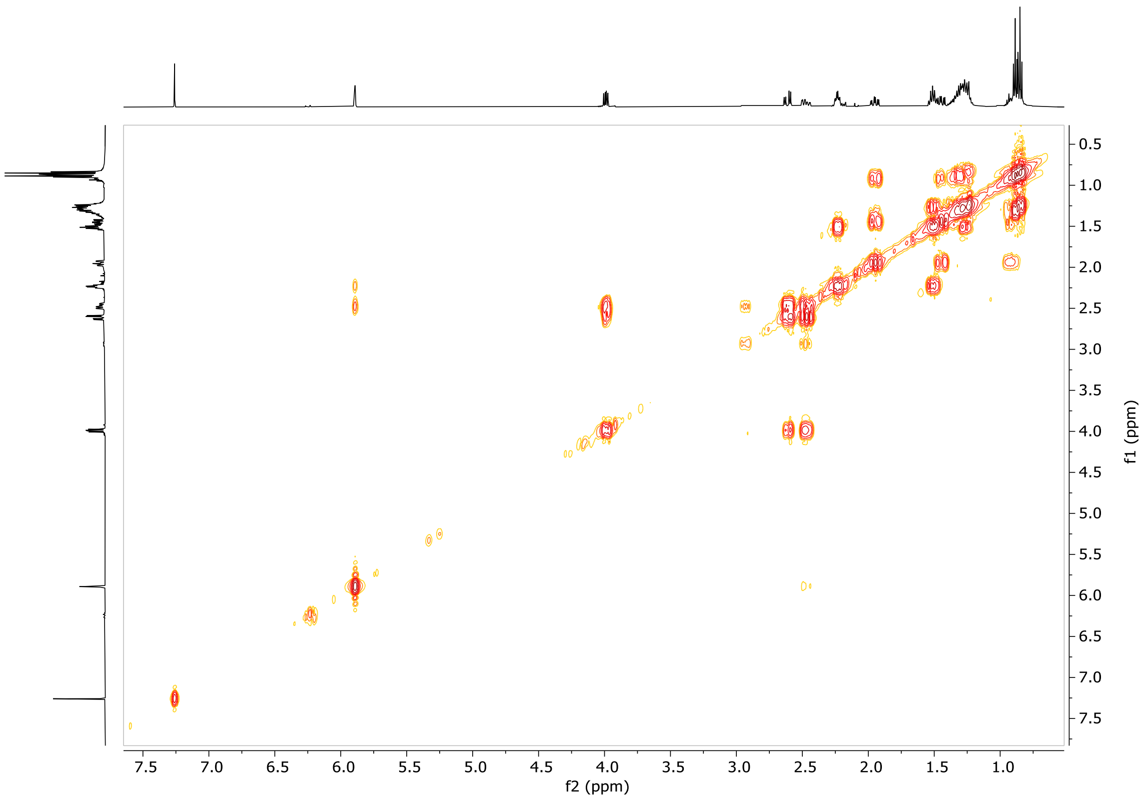

Figure S4. COSY spectrum of compound **1** (CDCl3, 500 MHz)

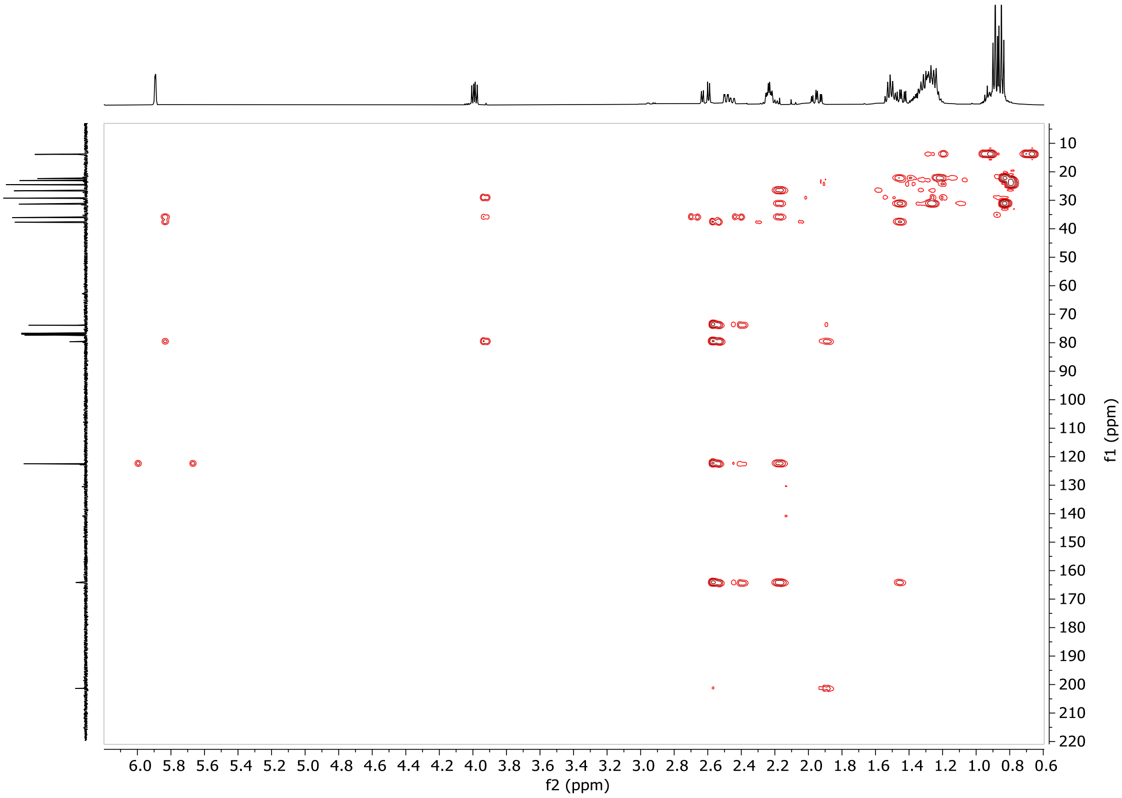

Figure S5. HMBC spectrum of compound **1** (CDCl3, 500 MHz)

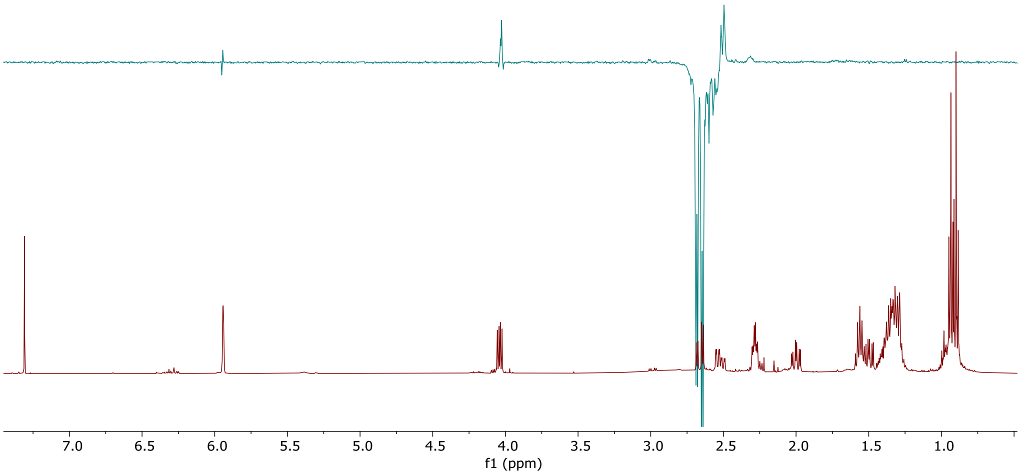

Figure S6. NOESY 1D spectrum of compound **1** (CDCl3, 500 MHz)

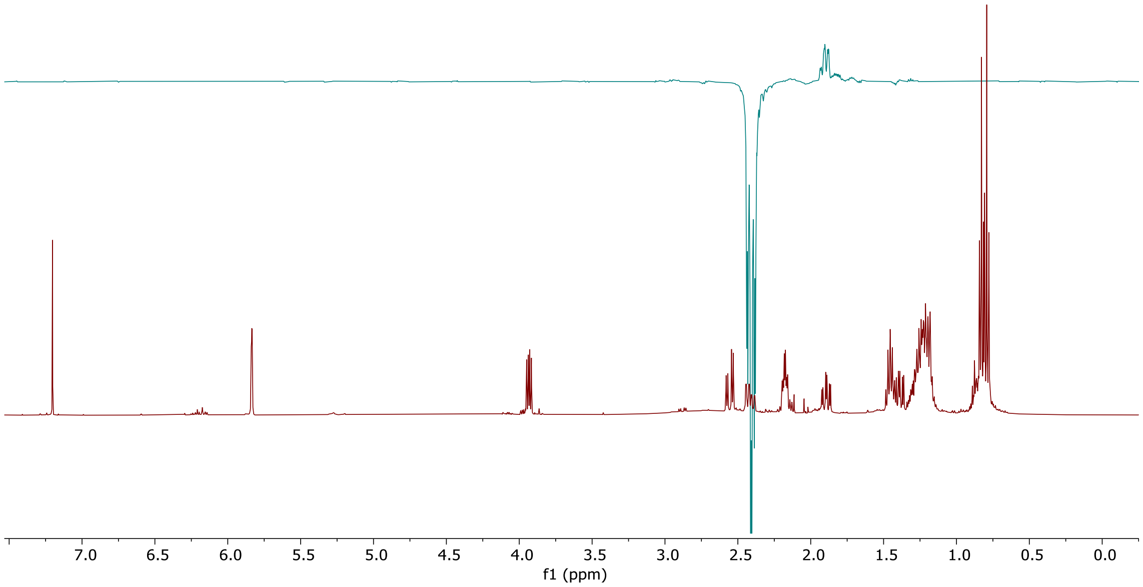

Figure S7. NOESY 1D spectrum of compound **1** (CDCl3, 500 MHz)

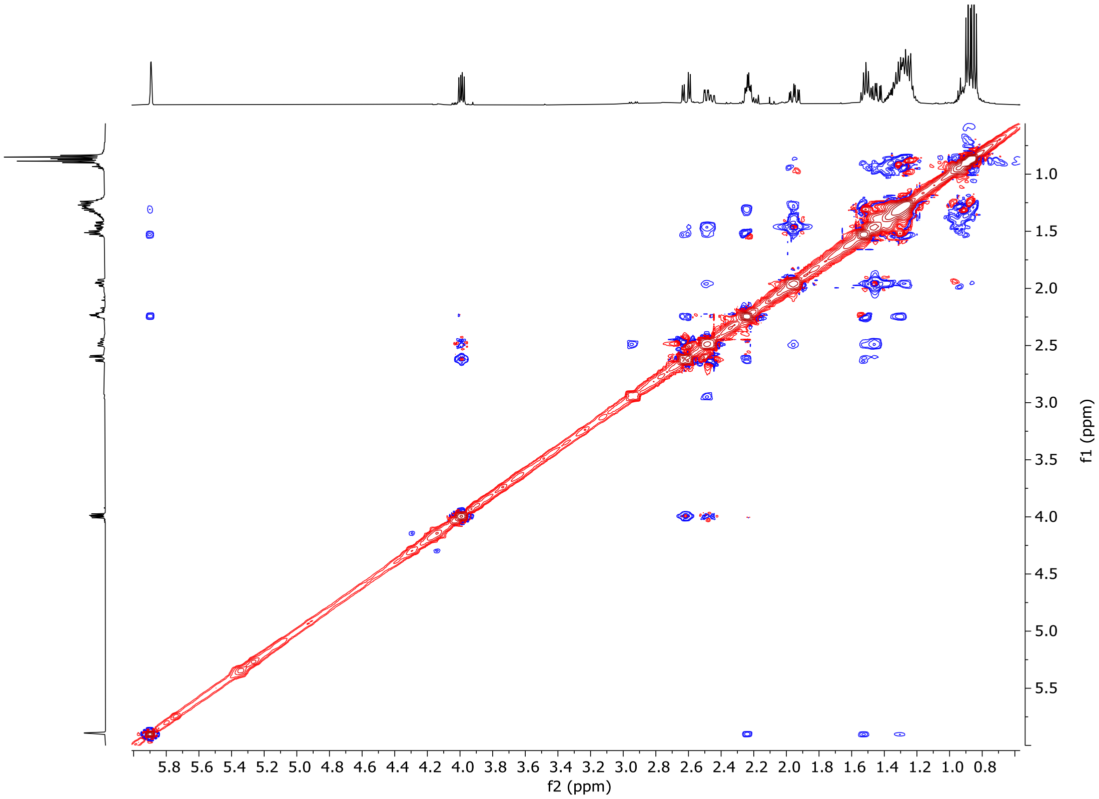

Figure S8. NOESY 2D spectrum of compound **1** (CDCl3, 500 MHz)

Elemental Composition Report **Page 1**

Tolerance = 5.0 PPM / DBE: min = -1.5, max = 200.0
Element prediction: Off

Monoisotopic Mass, Odd and Even Electron Ions
5 formula(e) evaluated with 1 results within limits (all results (up to 1000) for each mass)
Elements Used:
C: 15-15 H: 26-26 O: 0-4 Na: 0-2
Rodney (RL -H6D) E/I 70 ev Temp F 250 C
H14116-Rodney (RL -H6D) E I 70 ev Temp F 250 C 68 (3.320) Sm (Mn, 2x3.00)

Magnet EI+
1.12e+002

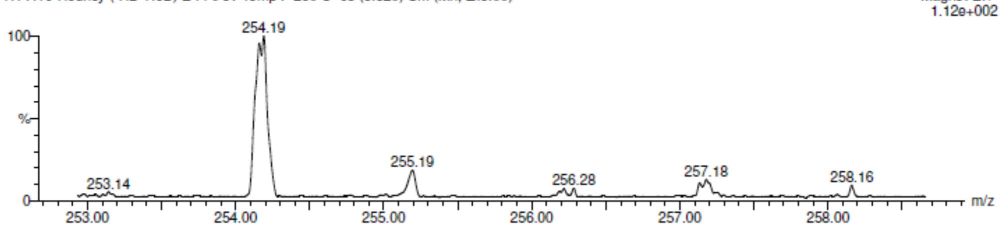

Minimum:	100.00				-1.5	
Maximum:	100.00		5.0	5.0	200.0	
Mass	RA	Calc. Mass	mDa	PPM	DBE	Formula
254.1888	100.00	254.1882	0.6	2.4	3.0	C15 H26 O3

Figure S9. HREIMS spectrum of compound **1**

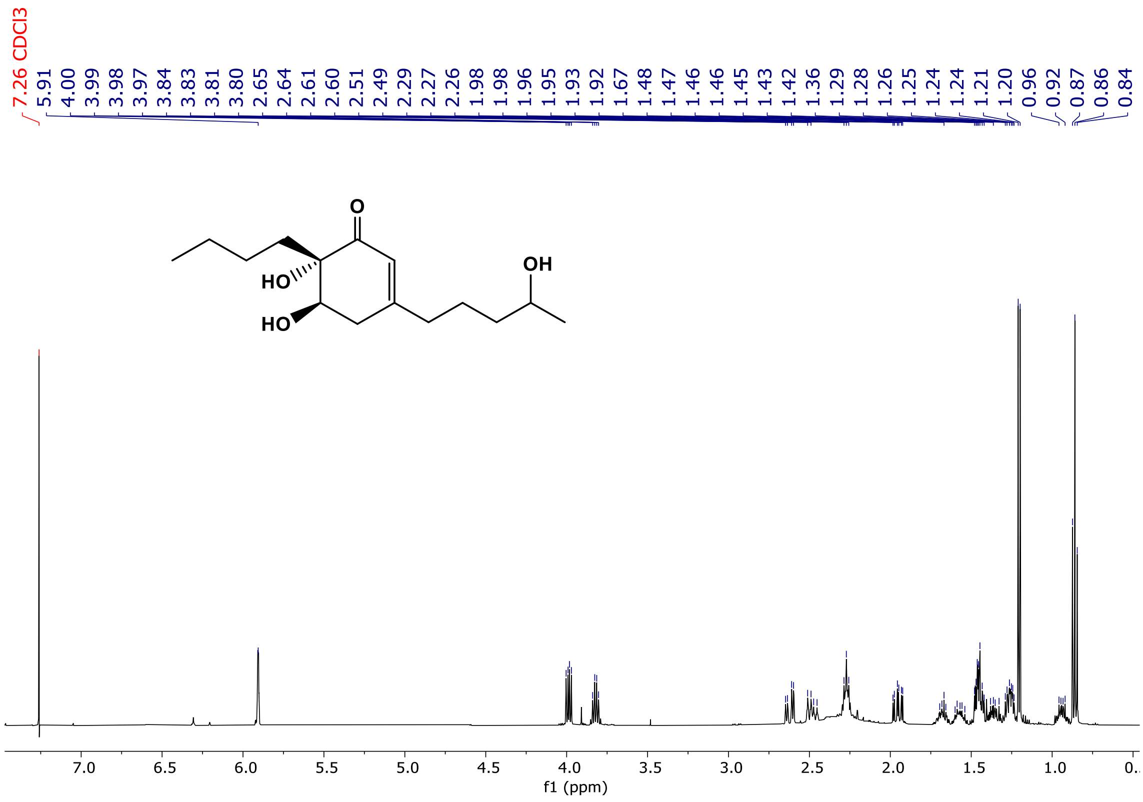

Figure S10. ^{1}H-NMR spectrum of compound **2** (CDCl3, 500 MHz)

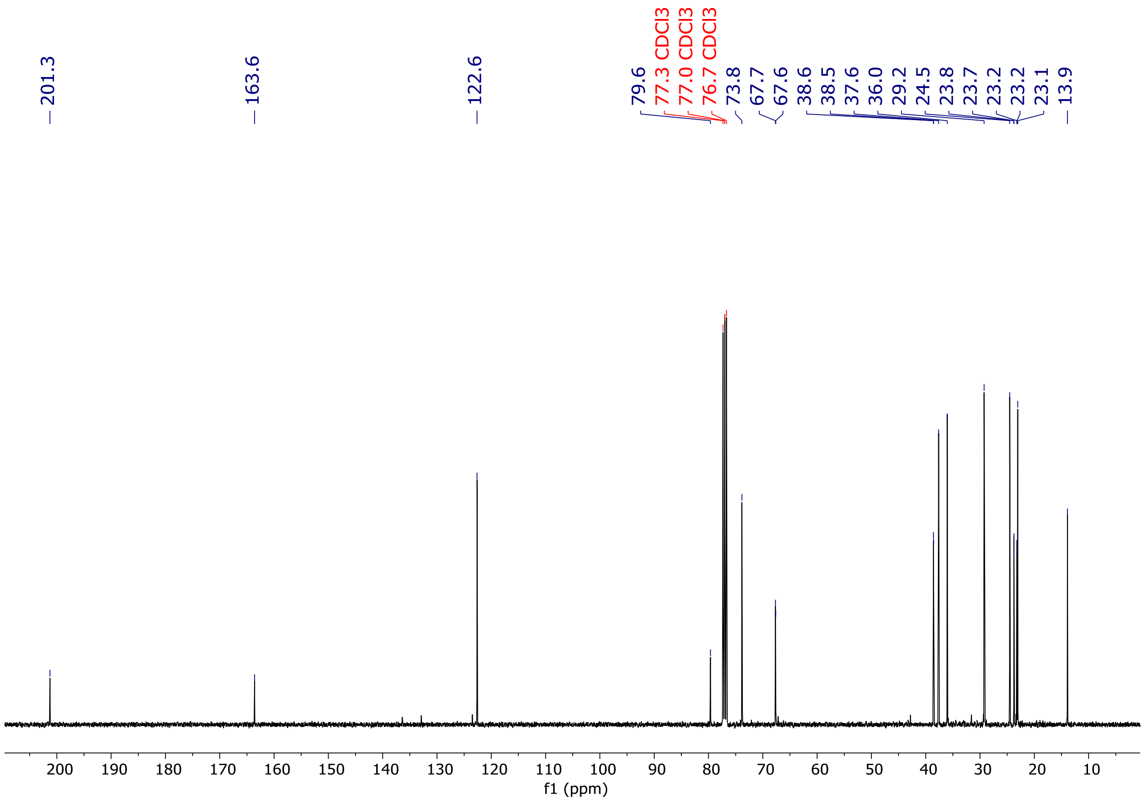

Figure S11. ^{13}C-NMR spectrum of compound **2** (CDCl3, 125 MHz)

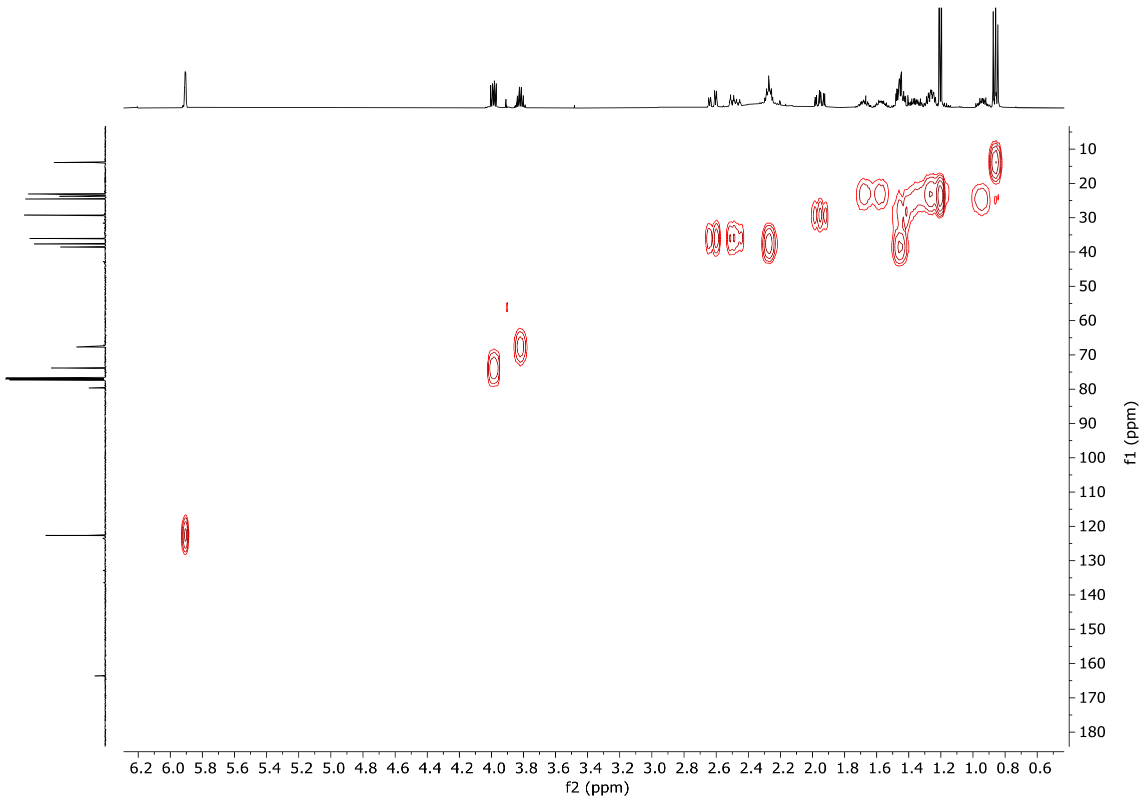

Figure S12. HSQC spectrum of compound **2** (CDCl3, 500 MHz)

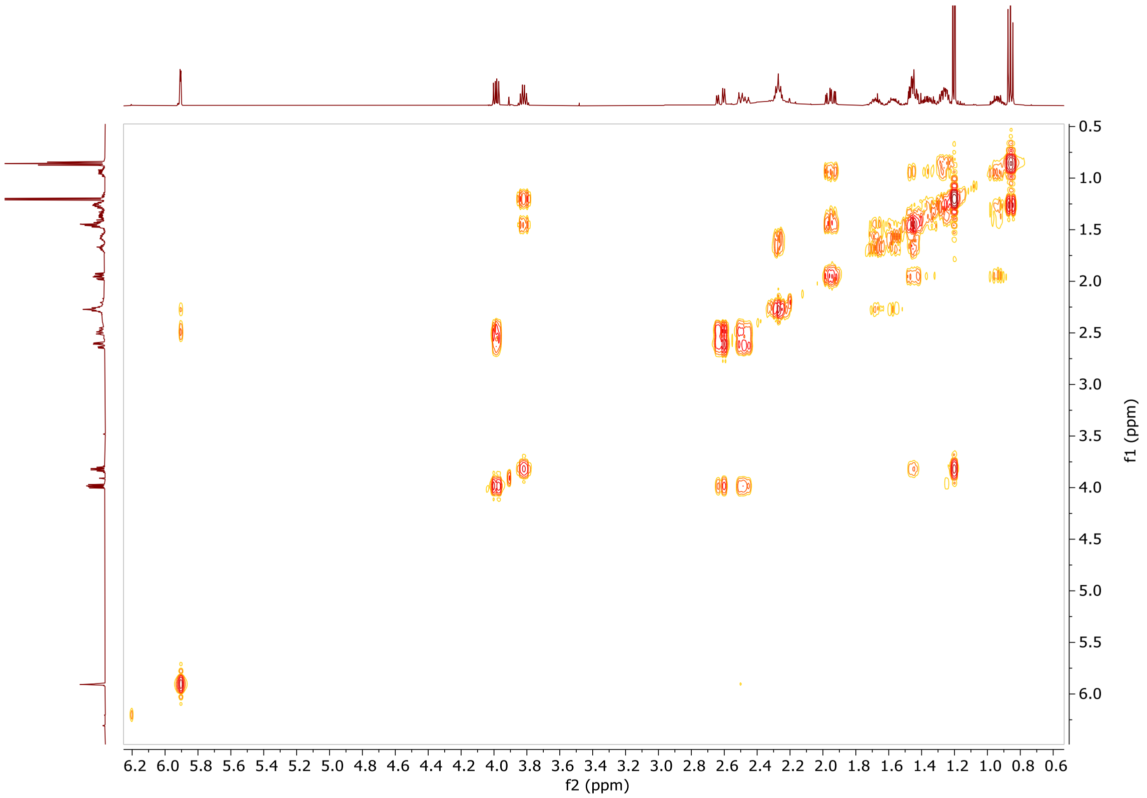

Figure S13. COSY spectrum of compound **2** (CDCl3, 500 MHz)

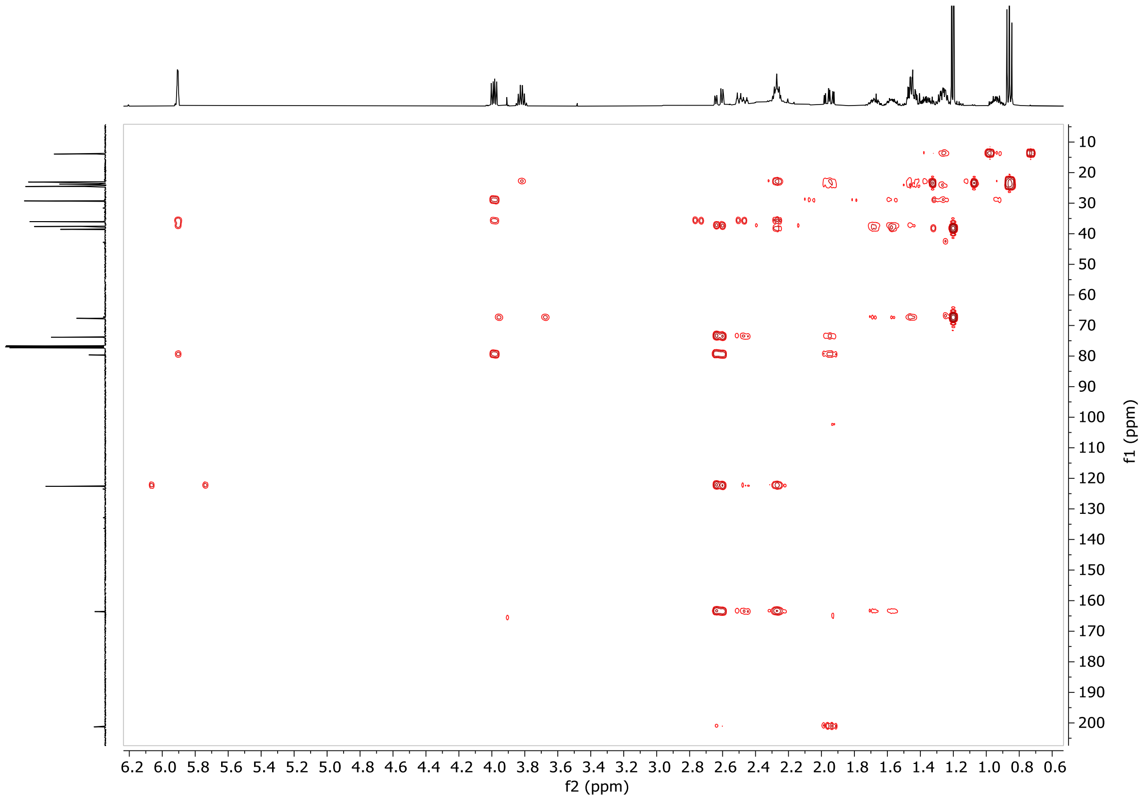

Figure S14. HMBC spectrum of compound **2** (CDCl3, 125 MHz)

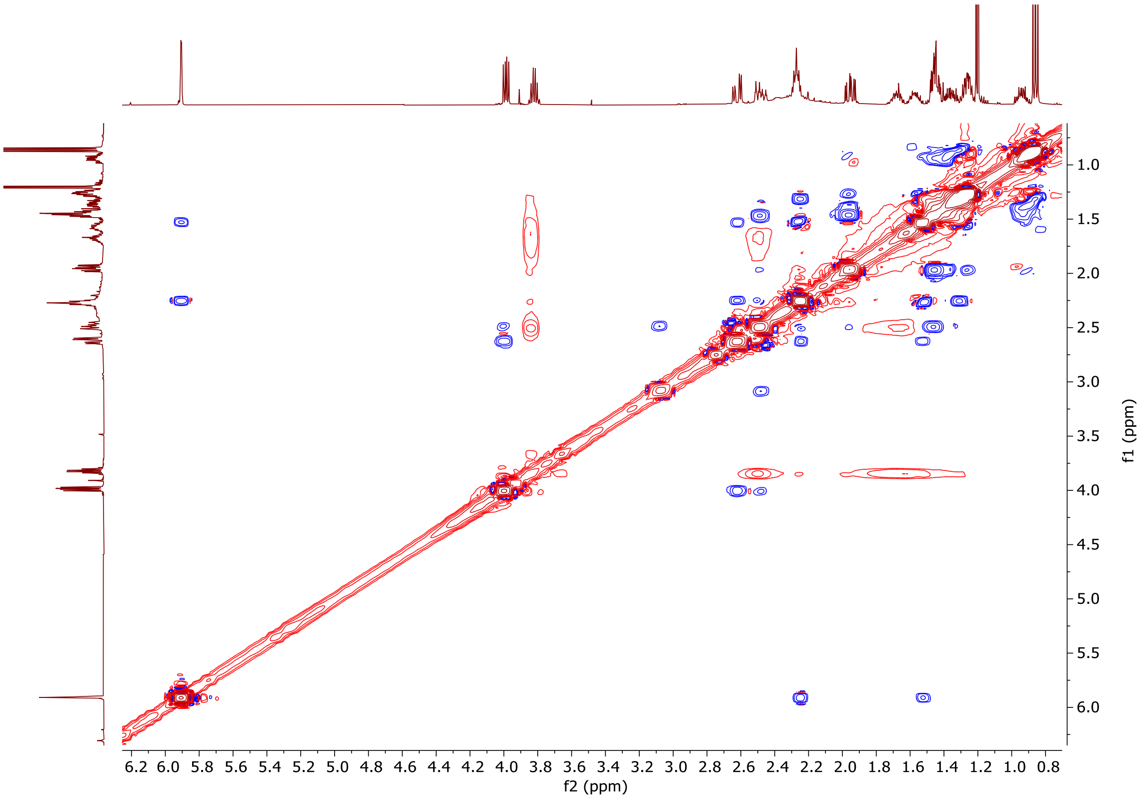

Figure S15. NOESY 2D spectrum of compound **2** (CDCl3, 500 MHz)

Elemental Composition Report **Page 1**

Multiple Mass Analysis: 2 mass(es) processed
Tolerance = 5.0 PPM / DBE: min = -1.5, max = 200.0
Element prediction: Off

Monoisotopic Mass, Odd and Even Electron Ions
10 formula(e) evaluated with 1 results within limits (all results (up to 1000) for each mass)
Elements Used:
C: 15-15 H: 26-26 O: 0-4 Na: 0-2
Rodney (RL-H6-17B) E/I 70 ev Temp F 250 C
H14123-Rodney (RL-H6-17B) E I 70 ev Temp F 250 C 117 (5.712)

Magnet EI+
6.37e+002

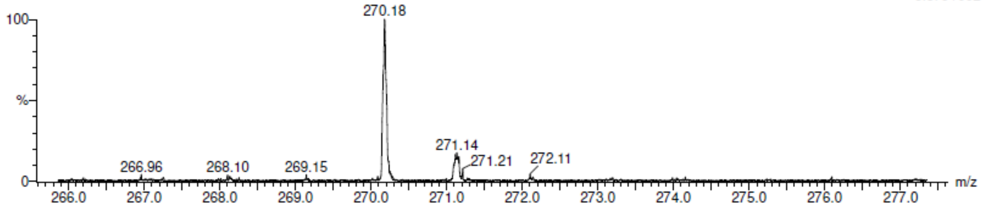

Minimum:	96.00				-1.5	
Maximum:	100.00		5.0	5.0	200.0	
Mass	RA	Calc. Mass	mDa	PPM	DBE	Formula
270.1793	100.00	---				
270.1823	96.23	270.1831	-0.8	-3.0	3.0	C15 H26 O4

Figure S16. HREIMS spectrum of compound **2**
